# Gonadal Function and Its Evolution in 46,XX Testicular/Ovotesticular DSD

**DOI:** 10.1210/clinem/dgaf603

**Published:** 2025-11-03

**Authors:** Margherita Sepich, Silvano Bertelloni, Nina Tyutyusheva, Angela Lucas-Herald, Inas Mazen, Martine Cools, Ruben Van Paemel, Şükran Poyrazoğlu, Olaf Hiort, Ulla Döhnert, Uta Neumann, Franziska Phan-Hug, Navoda Atapattu, Sumudu Seneviratne, Renata Markosyan, Rodolfo Rey, Sofia Suco, Federico Baronio, Corina Lichiardopol, Gilvydas Verkauskas, Marianna Stancampiano, Gianni Russo, Daniel Konrad, Nina Lenherr-Taube, Sabine Hannema, Gabriella Gazdagh, Diego Peroni, Syed Faisal Ahmed

**Affiliations:** Pediatrics Unit, Endocrinology Section, University of Pisa, Pisa University Hospital, Pisa 56126, Italy; Pediatrics Unit, Endocrinology Section, University of Pisa, Pisa University Hospital, Pisa 56126, Italy; Pediatrics Unit, Endocrinology Section, University of Pisa, Pisa University Hospital, Pisa 56126, Italy; Developmental Endocrinology Research Group, Royal Hospital for Children, University of Glasgow, Glasgow G51 4TF, UK; Department of Clinical Genetics, Human Genetics and Genome Research Division, National Research Centre, Cairo 11747, Egypt; Department of Paediatric Endocrinology, Ghent University Hospital, Ghent 9000, Belgium; Department of Internal Medicine and Paediatrics, University of Ghent, Ghent 9000, Belgium; Department of Paediatric Endocrinology, Ghent University Hospital, Ghent 9000, Belgium; Department of Internal Medicine and Paediatrics, University of Ghent, Ghent 9000, Belgium; İstanbul University, İstanbul Faculty of Medicine, Department of Pediatrics, Division of Pediatric Endocrinology, İstanbul 34893, Turkey; Division of Pediatric Endocrinology and Diabetes, Department of Pediatrics, University of Lübeck, Lübeck 23562, Germany; Division of Pediatric Endocrinology and Diabetes, Department of Pediatrics, University of Lübeck, Lübeck 23562, Germany; Centre for Chronic Sick Children, Department of Paediatric Endocrinology and Diabetology, Charité Universitätsmedizin Berlin, Berlin 133353, Germany; Paediatric Endocrinology, EHC-Morges, Morges 1110, Switzerland; Service of Endocrinology, Diabetology and Metabolism, Lausanne University Hospital, Lausanne 1011 Switzerland; Paediatric Endocrinology, Lady Ridgeway Hospital for Children, Colombo 00800, Sri Lanka; Department of Paediatrics, Faculty of Medicine, University of Colombo, Colombo 08, Sri Lanka; Yerevan State Medical University, Muratsan University Hospital, Clinic of Endocrinology, Yerevan 0025, Armenia; Centro de Investigaciones Endocrinológicas ‘Dr. César Bergadá’ (CEDIE), CONICET—FEI—División de Endocrinología, Hospital de Niños Ricardo Gutiérrez, Buenos Aires C1425EFD, Argentina; Centro de Investigaciones Endocrinológicas ‘Dr. César Bergadá’ (CEDIE), CONICET—FEI—División de Endocrinología, Hospital de Niños Ricardo Gutiérrez, Buenos Aires C1425EFD, Argentina; Pediatric Unit, IRCCS Azienda Ospedaliero-Universitaria di Bologna, Bologna 40138, Italy; Endocrinology, University of Medicine and Pharmacy Craiova, Craiova 200349, Romania; Children's Hospital, Vilnius University Hospital Santariskiu Klinikos, Vilnius 03101, Lithuania; Department of Pediatrics, Endocrine Unit, Scientific Institute San Raffaele, Milan 20132, Italy; Department of Pediatrics, Endocrine Unit, Scientific Institute San Raffaele, Milan 20132, Italy; Department of Pediatric Endocrinology and Diabetology and Children's Research Center, University Children's Hospital, University of Zurich, Zürich 8032, Switzerland; Department of Pediatric Endocrinology and Diabetology and Children's Research Center, University Children's Hospital, University of Zurich, Zürich 8032, Switzerland; Department of Pediatric Endocrinology, Amsterdam UMC Location Vrije Universiteit Amsterdam Emma Children's Hospital, Amsterdam 1081, The Netherlands; Amsterdam Gastroenterology Endocrinology Metabolism, Amsterdam HV 1081, The Netherlands; Amsterdam Reproduction and Development, Amsterdam 1105 AZ, The Netherlands; Wessex Clinical Genetics Service, University Hospital Southampton, Southampton SO16 5YA, UK; Department of Clinical and Experimental Medicine, Section of Pediatrics, University of Pisa, Pisa 56126, Italy; Developmental Endocrinology Research Group, Royal Hospital for Children, University of Glasgow, Glasgow G51 4TF, UK

**Keywords:** differences/disorders of sex development, ovotesticular DSD, testicular DSD, gonadal function, 46,XX DSD, I-DSD

## Abstract

**Context:**

There is scarce information on the natural history of gonadal function of testicular disorders/differences of sex development (T-DSD) and ovotesticular DSD (OT-DSD).

**Objective:**

To evaluate gonadal outcome in a large cohort of cases of T-DSD and OT-DSD.

**Methods:**

A total of 29 cases of T-DSD and 32 cases of OT-DSD were identified from 20 centers across 13 countries in the I-DSD Registry.

**Results:**

Male registration at birth occurred in 24 (83%) and 18 (56%) cases of T-DSD and OT-DSD, respectively. Of 42 cases registered as male, there were no cases of sex reassignment, while of 17 cases registered as female, 2 cases of T-DSD were reassigned within the first year of life. In male infants, stretched penile length (SPL) was <5th centile in 11/15 (73%) and similar in T-DSD and OT-DSD. However, in adolescence, median SPL in boys with OT-DSD (n, 5) and T-DSD (n, 4) was 5 cm (4.8, 7.5) and 9.5 cm (7.5, 12.5), respectively (*P* < .05). Of the 14 male and 4 female individuals who were aged >14 years, 7 (50%) and 2 (50%), respectively, had spontaneous puberty. In 8/9 (89%) male and 1/2 (50%) female adolescents and adults, serum gonadotropins were above reference range. However, in 8/9 (89%) males, serum testosterone was within the reference range. In 34 cases with available data, gonadal tumors had not been reported at a median age of 11.3 years (1 month, 35.5 years).

**Conclusion:**

In young adulthood, biochemical evidence of primary gonadal insufficiency is present in the majority of males and females with T-DSD and OT-DSD. In males with OT-DSD, micropenis may persist in young adulthood despite normal testosterone concentration.

46,XX testicular disorders/differences of sex development (T-DSD) is a very rare condition (with a reported prevalence of 1:20 000 of male newborns) characterized by the presence of a 46,XX karyotype, external genitalia ranging from typical male to ambiguous, and presence of testicular tissue in the gonads without any histologic evidence of ovarian tissue (1, 2). Ovotesticular DSD (OT-DSD) is an even rarer condition (with a birth prevalence of 1:100 000 live births) that is in most cases characterized by atypical genitalia in the presence of both functional ovarian and testicular tissue (as separate ovary and testis or as an ovotestis) (1, 3, 4). OT-DSD can be associated with a wide range of karyotypes including 46,XX and 46,XX/46,XY, and perhaps 46,XY and 45,X/46,XY (3, 5, 6). The most common genetic cause of these conditions is a translocation of *SRY* on the X chromosome or on an autosome, but several cases, especially in OT-DSD, have been reported to be associated with variants in *SOX3, SOX9, NR5A1, WT1, SOX10, WNT4,* and *RSPO1* (3, 7). As all known genetic causes of 46,XX T-DSD, apart from *SRY* translocation can also be associated with 46,XX OT-DSD, it is likely that these 2 conditions are part of a phenotypic spectrum within the same genetic entity (7). As there are no specific clinical practice guidelines for these conditions (8), the initial management approach has followed the general guidelines for DSD (9). However, there are several issues that remain challenging, and these include the identification of the gonadal tissue and the prediction of its functional evolution (1, 5, 10, 11). The international DSD Registry (I-DSD) has been shown to be a valuable resource for a wide range of rare conditions associated with a DSD (12) and the objective of the current study was to understand the natural history of gonadal function in cases of 46,XX T-DSD and OT-DSD that had been recruited to I-DSD.

## Methods

**Figure 1.**
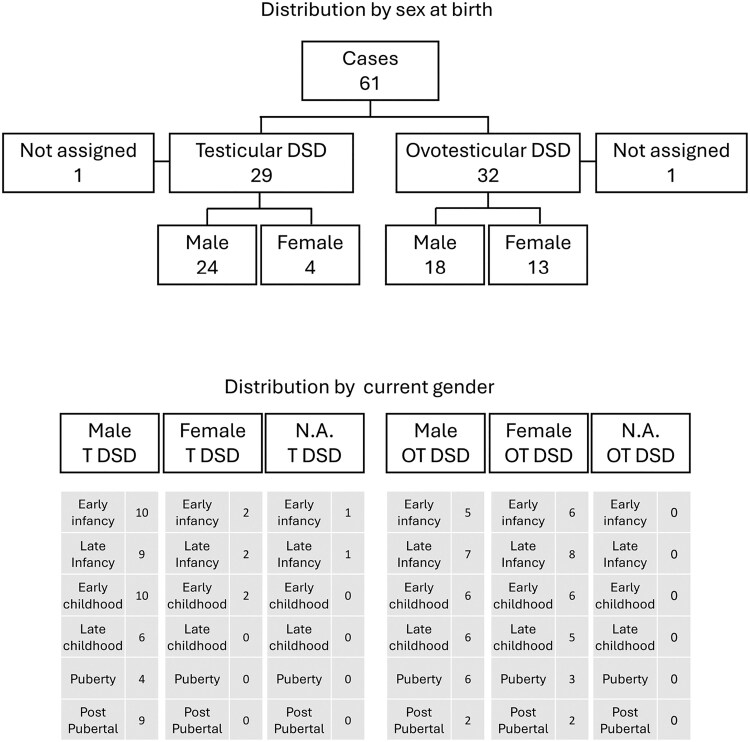
Distribution of cases based on diagnosis and sex registered at birth. Distribution of cases based on diagnosis, current gender (N.A. = Not Assigned) and number of available assessments in each age group. Early infancy (0 days to 6 months); late infancy (6 months to 2 years); early childhood (2 years to 7 years); late childhood (7 years to 12 years); adolescence (12 years to 16 years); adult (over 16 years).

The I-DSD Registry on the SDMregistries platform (https://sdmregistries.org/) was used to identify 46,XX cases of T-DSD or OT-DSD, and centers with eligible cases were invited to participate in the study. Centers were then asked to update the age-specific DSD dataset in I-DSD at ages closest to birth, 1 year, 5 years, 10 years, and 15 years (13). The retrospective dataset for the cases was then categorized into age categories: early infancy (0 days to 6 months); late infancy (6 months to 2 years); early childhood (2 to 7 years); late childhood (7 to 12 years); adolescence (12 to 16 years); adult (older than 16 years) (Fig. 1). The external masculinization score (EMS) was calculated based on description of the external genitalia (14). Stretched penile length was assessed using the reference data reported by Tomova et al (15). To assess gonadal function, locally measured values for serum luteinizing hormone (LH), follicle-stimulating hormone (FSH), testosterone, and anti-Müllerian hormone (AMH) were collected and compared to reference ranges (16-19). Those individuals who had undergone bilateral gonadectomy or who were on hormone replacement or puberty blockers were excluded from the analysis of the biochemical data. The data were described as medians and ranges and comparison between groups was performed using Mann-Whitney U test, Chi-squared test and odds ratio contingent table with IBM SPSS Statistics (version 29.0.2.0). I-DSD is an international database on the SDMregistries platform that collects pseudonymized information and is approved by the National Research Ethic Service in the United Kingdom as a research database of information that is collected as part of routine clinical care.

## Results

### Recruitment Data

Of the 27 centers with 102 eligible cases, from 13 countries in 4 continents, 20 agreed to participate, with a total of 61 cases and a median number of 2 cases per center (range 1, 12). Of these 61 cases, 29 (48%) had a reported diagnosis of T-DSD and 32 (52%) had a diagnosis of OT-DSD. In the 22 cases where the information was available, the median age at diagnosis was 5 months (birth, 55 years). The median age at last assessment was 11 years (1 month, 43.1 years).

### Sex Assignment and EMS

At birth, of the 29 cases with T-DSD, 24 (83%) were registered as male and 4 (14%) as female. Of the 32 cases with OT-DSD, 18 (56%) were registered as male and 13 (40%) as female. Two cases had delayed sex registration; one case of T-DSD was registered male and one case of OT-DSD was registered female, both within the first year of life. Of the 42 cases registered as male, there were no cases of sex reassignment, while of the 17 cases registered as female, 2 cases of T-DSD, 1 with an EMS of 3 and the other with an EMS of 7, were reassigned within the first year of life, to male sex and “undecided,” respectively. The number of cases born before 2006 and after 2006 were 24 and 37, respectively and the odds ratio of being assigned as female in those born after 2006 compared to those born before 2006 was 1.6 (lower 95% CI 0.5; upper 95% CI 5.6). At the time of the study, the median current age of individuals with T-DSD who were registered male and female was 18.8 years (2.1, 56) and 10.7 years (3.8, 11), respectively. For OT-DSD, the median male and female current ages were 15.1 years (5.7, 48.2) and 17.1 years (6.3, 40.9), respectively. In the 32 cases of T-DSD and OT-DSD where EMS could be calculated at presentation, the median EMS was 7.25 (1, 11.5) in male (n, 20) compared to 3 (3, 7) in the female cases (n, 11) (*P* < .05). There was one additional case without sex assignment who had an EMS of 3.

### Genetics

Of the 61 cases, details of genetic investigations were available in 34 (56%). Of these, 13 (38%) were positive for SRY. In the remainder, 3 had a SF1 variant (missense, R92W) and there was 1 case each of an X chromosome duplication ([Xp22.33(877 796 × 2, 896734_1383266 × 3, 1404739 × 2] reported as VUS), *KANK1* deletion (chr 9), *DMRT1* duplication (arr[GRCh37] 9p24.3[845,893_896,518 × 3]) and *SOX3* duplication (arr[hg19] Xq27.1[139,541,737-140,043,863 × 3]). All SRY positive cases were T-DSD while, of the 21 SRY negative cases, 10 were T-DSD and 11 OT-DSD.

### Pubertal and Penile Growth

**Figure 2.**
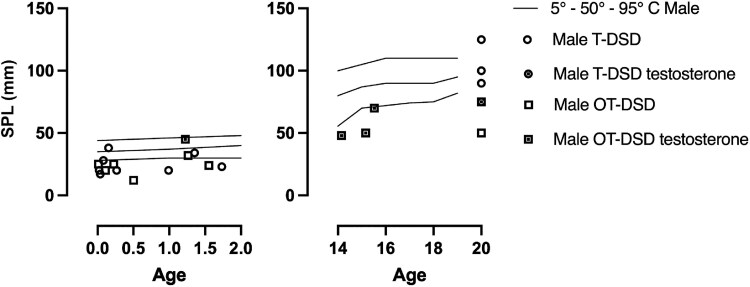
Distribution of stretched penile length (SPL, mm) in male subjects with reference ranges for the specific age periods: on the left, in early and late infancy (from birth to 2 year) and on the right, in adolescence and adulthood (from 12 to over 20 years old). Open circles represent T-DSD (testicular DSD) without testosterone supplementation; Open box represents OT-DSD (ovotesticular DSD) without testosterone supplementation, Closed circle represents T-DSD (testicular DSD) on testosterone supplementation, Closed box represents OT-DSD (ovotesticular DSD) on testosterone supplementation. The 5th, 50th, 95th centiles for SPL are based on data from Tomova et al (15).

Of the 18 males over 14 years of age, 14 had information on pubertal development. Of these, 7 (50%) had spontaneous onset of puberty while 7 (50%) required pubertal induction (2 of them had gonadal removal). The EMS in infancy was available in 7 of these 14 cases. Of these 7 cases, 4 had spontaneous puberty and a median EMS at initial presentation of 10.5 (6, 11), while 3, with a median EMS of 7.5 (7, 7.5), required pubertal induction (one of them underwent gonadal removal). Of the 4 female individuals who were over 14 years old at the time of the study, 2 had spontaneous puberty while 2 required pubertal induction. In males in early and late infancy (n, 15), the stretched penile length (SPL) was <5th centile in 11 (73%). Only one case was recorded to receive testosterone supplementation during early infancy and his SPL was reported to be within the normal range during that period. In the early and late infancy age groups, in male cases with OT-DSD (n, 7), the median SPL was 2.5 cm (1.2, 4.5) and in male cases with T-DSD (n, 8), the median SPL was 2.2 cm (1.7, 3.8) (NS). In the adolescent and adult groups (n, 9), there were 6 male cases with a SPL that was < 5th centile, and of these 4 were on testosterone supplementation (Fig. 2). Among these 9 cases, there were 5 cases of OT-DSD (n, 5) with a median SPL of 5 cm (4.8, 7.5) and 4 cases of T-DSD with a median SPL of 9.5 cm (7.5, 12.5) (*P* < .05).

### Gonadal Histology and Tumor Outcomes

In the 24 cases where information was available in early infancy, there were a total of 9 ovotestes, 6 ovaries, and 18 testes; of 15 gonads (in 8 cases) no information on the gonadal structure was available. Anatomical distribution of the gonads is described in Table 1. Histological information obtained through biopsies and/or gonadectomies was available for 27 gonads in a total of 29 cases (20 OT-DSD and 9 T-DSD). The following findings were reported in the left gonad: ovarian and testicular tissue (n, 8; all OT-DSD); only ovarian tissue (n, 6; all OT-DSD); only testicular tissue (n, 4; 1 T-DSD, 3 OT-DSD); Leydig cell hypoplasia (n, 1, T-DSD); partial dysgenetic testicular tissue (n, 2, both T-DSD), dysgenetic testicular tissue (n, 4; 1 T-DSD, 3 OT-DSD); immature testicular tissue (n, 1, OT-DSD). The following findings were reported in the right gonad: ovarian and testicular tissue (n, 7; all OT-DSD); only ovarian tissue (n, 4; all OT-DSD); only testicular tissue (n, 2; both OT-DSD); Leydig cells hypoplasia (n, 1; T-DSD); partial dysgenetic testicular tissue (n, 4; all T-DSD), dysgenetic testicular tissue (n, 4; 3 T-DSD, 1 OT-DSD); immature testicular tissue (n, 2; both OT-DSD); partial dysgenetic ovarian tissue (n, 1; OT-DSD). In another 3 cases, the diagnosis of OT-DSD had been reached without any histological evidence of ovarian tissue. Of these 3 cases, ovarian tissue had been suspected in one on ultrasound-based appearance of ovarian cysts but biopsy had only revealed testicular tissue; in another case an ovary was identified on ultrasound and histological evidence was not pursued, and in the last case, although only testicular tissue was observed at biopsy, a diagnosis of OT-DSD was reached because of a low EMS of 3 out of 12 and the presence of a normal uterus. A total of 14 individuals underwent gonadal removal, 10 unilateral and 4 bilateral. Of the 10 out of 14 where a reason for gonadectomy was reported, 6 were for gender conforming, 2 for gender conforming and tumor risk mitigation, and 2 for tumor risk mitigation only. Data on development of gonadal tumors was available in 34 cases (17 T-DSD, 17 OT-DSD) and the median age at last assessment of these cases was 11.3 years (1 month to 35.5 years) with 4 cases being over 20 years old. The presence of tumors was not reported in any of these cases.

**Table 1.** Distribution of gonads in the patients in early infancy

Gonad location	Ovary		Testis		Ovotestis		Unknown	
	Left	Right	Left	Right	Left	Right	Left	Right
Labioscrotal	1	0	5	7	0	0	2	2
Impalpable	3	2	0	1	4	3	3	2
Inguinoscrotal	0	0	2	3	1	1	2	1
Unknown	0	0	0	0	0	0	1	2
Total	4	2	7	11	5	4	8	7

### Müllerian Phenotype

Information on Müllerian development was available in 30 cases that included 11 cases of T-DSD and 19 cases of OT-DSD. Of these 30, a normal uterus was present in 8 (27%) cases of OT-DSD. A rudimentary uterus was present in 6 (20%) cases that included 2 cases of T-DSD and 4 cases of OT-DSD. A hemi-uterus was reported in 3 (10%) cases and this included 1 case of T-DSD and 2 cases of OT-DSD. Müllerian remnants were reported in 2 (7%) cases of OT-DSD. Lastly, no Müllerian structures were reported in 11 (37%) cases, which included 8 cases of T-DSD and 3 cases of OT-DSD. In summary, some form of Müllerian structures was present in 3 out of 11 (27%) cases of T-DSD and 16 out of 19 (84%) cases of OT-DSD (*P* = .01).

### Changes in Gonadotropins

**Figure 3.**
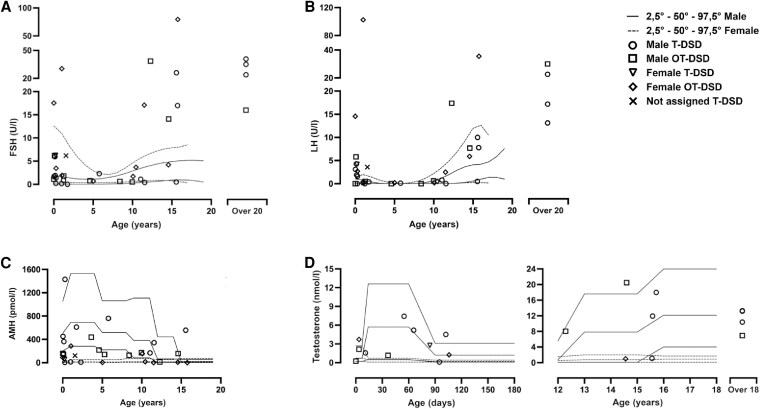
Distribution of biochemical data with specific reference ranges for males (solid line) and females (broken line). Open circles, Male T-DSD (testicular DSD); Open box, Male OT-DSD (ovotesticular DSD); Open triangle, Female T-DSD (testicular DSD); Open diamond, Female OT-DSD (ovotesticular DSD); Cross, Not assigned T-DSD (testicular DSD). (A) FSH (UI/L); (B) LH (UI/L); (C) AMH (pmol/L); (D) Total testosterone (nmol/L) on the left in early infancy (from birth to 6 months) and on the right in adolescence and in the adult period (from 12 to over 20 years old).

Of the 11 males in early and late infancy in whom LH values were available, 5 (45%) had levels that were >97.5th centile (Table S1) (20). In the adolescent group of 5 males, 4 (80%) had an LH >97.5th centile. In the adult group which consisted of 4 males, all had an LH >97.5th centile. In the 5 females in early and late infancy who had LH measurements, 4 (80%) had an LH >97.5th centile. In the female adolescent group 1 of the 2 (50%) cases had an LH >97.5th centile. The single case without sex assignment presented with an LH which was >97.5th centile for males and females (Fig. 3, Table S1) (20). FSH values in the 11 males in early and late infancy were >97.5th centile in 4 (36%). In the adolescent group of 5 males, 4 (80%) had an FSH of >97.5th centile. In the male adult group, all 4 (100%) cases had an FSH of >97.5th centile. In the 5 females in early and late infancy, FSH was >97.5th centile in 2 (40%). In the female adolescent group 1 out of 2 (50%) had an FSH >97.5th centile. The case without sex assignment had a FSH >97.5th centile for the male reference range but within the female reference range (6.2 UI/L) (Fig. 3).

### Changes in Testosterone

In early infancy, testosterone values in male cases were below the reference range in only 1 out of 8 (12%) cases. In the male adolescent group of 5 cases there was also only 1 (20%) who had testosterone levels below the reference range. In the male adult group, all 4 were within the reference range between 6.9 and 13.2 nmol/L. The 3 females in the early infancy group showed testosterone values above the female reference range (Fig. 3). In the adolescent group the single female case had values within the female reference range (Table S1) (20).

### Changes in AMH

AMH values were available in 15 male children and of these 8 (53%) had levels that were under the 2.5th centile for males (Fig. 3). In the 6 female children where levels were available, 4 (67%) had a AMH value that was above the normal female reference range. In all 6 cases, the AMH value was <50th centile for the male reference range and in 4 (67%) it was <2.5th centile. In the 3 adolescent and adult females where AMH values were available AMH was >2.5th centile in 2 cases (67%) (Fig. 3, Table S1) (20).

## Discussion

46,XX T-DSD and OT-DSD are very rare conditions. As such, their management can be very challenging (1, 8) and in counseling and decision making, the prognosis of long-term gonadal function is often taken into account. Most of the evidence is based on case reports and single-center experience and the I-DSD Registry offered the chance to investigate a large international cohort of these 2 DSDs.

All patients had a confirmed 46,XX karyotype and for those who had specifics on the genetics, 13 were *SRY* positive and 21 were *SRY* negative. In the *SRY* negative group, mutations involving *SF1*, X chromosome, *KANK1*, *DMRT1*, or *SOX3* were observed. At birth, the likelihood of being registered as male was greater and this was seen in those cases that were labeled as T-DSD as well as OT-DSD. At any rate, children with less virilization were more likely to be raised as female. An increased likelihood being registered to the female sex in the infants born after 2006 has been observed; this was contrary to a previous report of more cases of XY DSD being registered male (21). This finding may be related to a shift in improvements in the identification of ovarian tissue and placing a greater emphasis on the functional potential of ovarian tissue (9). The median age at the time of the study was 11 years, ranging up to the 40s, and in more than 95% of cases, sex assignment had occurred in early infancy or at birth. Gender dysphoria has previously been reported to have an incidence of 10% to 20% in those with a diagnosis of OT-DSD and who were initially raised as female (10). However, in the current series, gender incongruence did not occur in any of the case of this series; in the 2 individuals where sex reassignment was reported, this had been undertaken within the first year of life. In this series, a sizeable proportion of cases of T-DSD (14%) were raised as female, given that T-DSD is usually associated with strong virilization (22-24). This finding may have arisen due to a bias in the I-DSD Registry which has primarily focused on cases that have presented in childhood. Similarly, despite OT-DSD being described as far rarer than T-DSD, in our sample the OT-DSD group was larger than the T-DSD group and this may simply reflect the mix of cases in the I-DSD Registry. Those with minor signs of undervirilization, typically T-DSD, are likely to be identified in adulthood in a different clinical setting and would have been under-reported in I-DSD (25).

The analysis of the available biochemical data showed that there was a gradual trend toward a deterioration in gonadal function in both T-DSD and OT-DSD and this was primarily demonstrated by the rise in gonadotropins from childhood to adulthood in both males and females. On the other hand, androgen production itself was not as profoundly affected in those raised as males, highlighting the potential for entering puberty spontaneously (6, 26, 27) despite a biochemical picture of hypergonadotropic hypogonadism (22). Indeed, around half of those raised as male, or female, entered puberty without the need for sex hormones. The finding of AMH values below average in almost all boys in childhood also confirmed that Sertoli cell function in those raised as male was suboptimal. The low AMH may also explain the finding of a Müllerian structure in 3 cases of T-DSD and contrary to the reports in the literature (22, 28, 29).

It should be underlined that the biochemical data were collected from several centers over a long period of time using different assays and this could represent a bias. While azoospermia is universally encountered in men with T-DSD, the ovarian tissue in OT-DSD has fertility potential as would the testicular tissue in cases OT-DSD with a Y-chromosome (30). There is, therefore, a need for further longer-term studies to explore fertility outcomes in this cohort. Despite biochemical data indicating androgen production, micropenis frequently occurred in males and was also present in individuals on testosterone supplementation. To our knowledge, this finding has not been reported before in cases of OT-DSD or T-DSD. Not only does it have a clinical and prognostic implication, but it may also shed some light on penile development, some of which may be androgen independent and may indeed depend on the balance between prenatal androgen and estrogen exposure (31, 32). Furthermore, the fact that OT-DSD cases had a significant lower SPL in the later stage of life also raises a question about the possibility that estrogen exposure at a critical timepoint, such as the masculinization programming window, may have a long-lasting inhibitory effect on phallic growth (33, 34).

Although the current report represents the largest international group of cases that have been studied, the lack of standardized follow-up and documentation of these cases was an obstacle in obtaining a thorough understanding of the natural history of this rare DSD. Several cases did not have a sufficient level of detail on the size of the genital tubercle, location of the urethral meatus or extent of labioscrotal fusion in early infancy to calculate the external genitalia score (EGS) (35) and in this situation, the EMS, which does not rely on this high level of granularity was found to be more informative, especially as it could also be applied across the age span.

**Figure 4.**
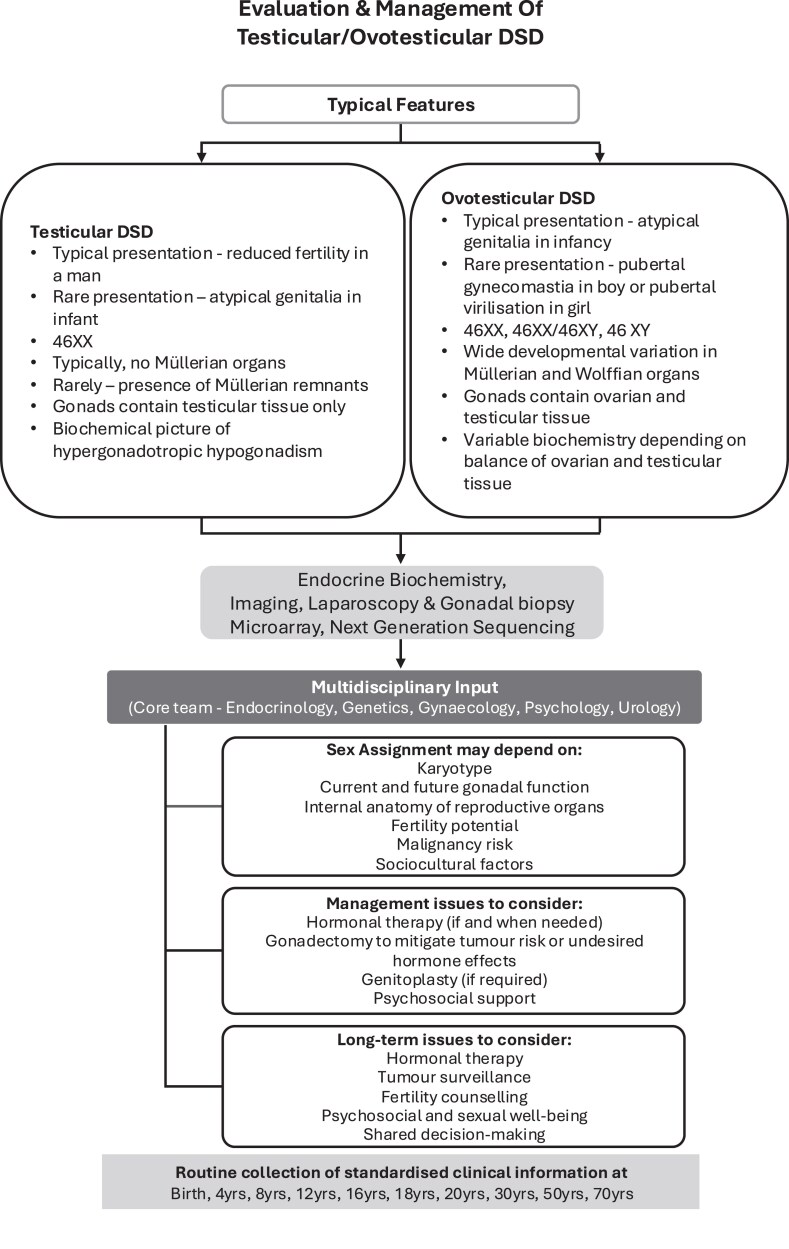
Summary of a pathway for evaluating and managing suspected cases of testicular and ovotesticular DSD.

The absence of gonadal tumors was not unexpected, given that the XX karyotype was an inclusion criterion. However, data on the histological structure of the gonads through biopsies or gonadectomies was only available for around half of the cases. Historically, the risk of gonadal tumors has also been reported as low in OT-DSD (36) but this risk has been questioned more recently when a third of cases at a single center were reported to have evidence of gonadal tumor (37). While the results of the current study are reassuring, the current group of cases is still relatively young for reporting on gonadal tumor as well as fertility outcomes, and to study these issues there remains a need for further standardized longitudinal studies. The current survey also highlighted that roughly half of the cases had not undergone detailed genetic investigations, and this may be related to the lack of availability or resources as well as a clear understanding of the value of genetic analysis in this group of conditions. Although the heterogeneous phenotype of T-DSD and OT-DSD preclude a standard approach that can be applied to all cases, there are, nevertheless, some general considerations that include the need for multidisciplinary input and standardized assessments at regular frequency that could be recommended for all suspected cases (13) (Fig.4).

In summary, the current data show that young people with conditions such as T-DSD and OT-DSD who present in early childhood can enter puberty of their own accord. However, long-term prospects for their gonadal function remain guarded. Micropenis in those raised as male is a common feature and may persist into adulthood despite androgen replacement, particularly in OT-DSD.

## Data Availability

All the relevant data underlying this article are available in the article and in its online supplementary material (20). Raw and derived data supporting the findings of this systematic review are available from the corresponding author on request.

## Abbreviations

AMH: anti-Müllerian hormone
DSD: disorders/differences of sex development
EMS: external masculinization score
FSH: follicle-stimulating hormone
LH: luteinizing hormone
OT-DSD: ovotesticular disorders/differences of sex development
SPL: stretched penile length
T-DSD: testicular disorders/differences of sex development
